# The Impact of Individual Factors on Careless Responding Across Different Mental Disorder Screenings: Cross-Sectional Study

**DOI:** 10.2196/70451

**Published:** 2025-07-31

**Authors:** Huawei Kuang, Lichao Zhu, Haonan Yin, Zihe Zhang, Biao Jing, Junwei Kuang

**Affiliations:** 1 School of Energy and Constructional Engineering, Shandong Huayu University of Technology, Dezhou, China; 2 Tencent (China), Shenzhen, China; 3 Paul Merage School of Business, University of California, Irvine, CA, United States; 4 Department of Industrial Engineering, School of Business Administration, South China University of Technology, No. 381 Wushan Road, Guangzhou, 510641, China, 86 18810889628

**Keywords:** careless response, digital health, online health screening, mental health, questionnaire

## Abstract

**Background:**

Online questionnaires are widely used for large-scale screening. However, careless responding (CR) from participants can compromise the reliability of screening outcomes. Prior studies have focused on the effects of individual and environmental factors on CR, but the effect of questionnaire type remains underexplored.

**Objective:**

This study investigates the individual factors influencing CR in online mental health screening and assesses how the effect of these factors varies across different psychological questionnaires.

**Methods:**

This study analyzed data from 24,367 participants across 4 questionnaires (PHQ-9 [Patient Health Questionnaire-9], PSS [Perceived Stress Scale], ISI [Insomnia Severity Index], and GAD-7 [Generalized Anxiety Disorder-7 Scale]). CR was defined as the proportion of items completed in less than 2 seconds per item. We used a multiple linear regression model to examine the effect of individual factors (sex, age, education, smoking, and drinking) on CR across 4 questionnaires. In addition, response times were visualized to identify patterns between careless and careful responders.

**Results:**

Females demonstrate lower levels of CR than males when completing the PHQ-9 (*β*=−.172, 95% CI −0.104 to −0.089; *P*<.001), PSS (*β*=−.234, 95% CI −0.162 to −0.14; *P*<.001), ISI (*β*=−.207, 95% CI −0.13 to −0.114; *P*<.001), and GAD-7 (*β*=−.177, 95% CI −0.108 to −0.093; *P*<.001). Older participants demonstrated lower levels of CR on the PHQ-9 (*β*=−.036, 95% CI −0.007 to −0.003; *P*<.001), ISI (*β*=−.036, 95% CI −0.007 to −0.003; *P*<.001), and GAD-7 (*β*=−.053, 95% CI −0.009 to −0.005; *P*<.001), but their age was unrelated to CR on the PSS. Interestingly, compared with participants with an associate-level education, those with a high education (bachelor’s, master’s, or doctoral degree) demonstrated higher levels of CR, especially those with a master’s degree (PHQ-9: *β*=.098, 95% CI 0.136 to 0.188; *P*<.001 and GAD-7: *β*=.091, 95% CI 0.125 to 0.178; *P*<.001). Smokers exhibited varied patterns, with current smokers demonstrating lower levels of CR on the PHQ-9 (*β*=−.022, 95% CI −0.064 to −0.016; *P*=.001) and GAD-7 (*β*=−.014, 95% CI −0.051 to −0.002; *P*=.03), whereas occasional smokers demonstrated higher levels of CR on the PSS (*β*=.019, 95% CI 0.010 to 0.050; *P*=.003) than nonsmokers. Drinkers demonstrated lower levels of CR than nondrinkers, with the strongest effect among occasional drinkers on the PHQ-9 (*β*=−.163, 95% CI −0.103 to −0.087; *P*<.001). Analysis of response times revealed that participants tended to spend less time on PHQ-9 and GAD-7 surveys, and CR on PSS and ISI surveys was characterized by skipping questions.

**Conclusions:**

The effect of individual factors on CR varies across questionnaire types. These findings offer valuable insights for questionnaire designers and administrators, highlighting the need for targeted intervention.

## Introduction

### Background

Mental illnesses, such as depression and anxiety, are associated with symptoms including low mood, difficulty concentrating, and a diminished interest in life, profoundly affecting both individuals’ well-being and societal stability [1,2]. Psychological issues have become increasingly prevalent worldwide. The COVID-19 pandemic has exacerbated this trend, leading to a sharp rise in mental health cases [3]. According to the World Health Organization (WHO), nearly 1 billion people globally endure from mental disorders, with cases of anxiety and depression increasing by 25% following the pandemic [4]. However, due to mental illnesses being stigmatized in contexts, particularly depression and anxiety, individuals often hesitate to disclose their conditions to health care providers [5]. This reluctance results in a significant number of unreported and untreated cases, ultimately exacerbating the overall disease burden [6,7]. Consequently, implementing effective mental health screening is essential for early detection and prevention [8,9].

Mental health screening questionnaires are standardized tools designed to assess an individual’s mental health status through a series of structured questions. Traditionally, these questionnaires were distributed by administrators to participants for completion, after which they were collected and analyzed. However, this method is often hindered by inefficiencies in distribution, data collection, and analysis. The emergence of internet technology has revolutionized this process by introducing online psychological screening questionnaires, which offer a more efficient and scalable alternative. Online questionnaires can improve the recognition of mental illness and increase patients’ opportunities to receive digital mental health interventions [10]. Compared to paper questionnaires, they are more easily accepted by respondents in modern mental health screening [11]. Despite their advantages, a significant limitation of online questionnaires is the increased risk of careless responding (CR), where participants may fail to read or fully engage with the items [12-14]. This can lead to data that inaccurately reflects their true mental health status [15], thus undermining the validity of the results and subsequent psychological interventions. Consequently, identifying the factors that influence CR and improving response validity has become a critical priority.

### Related Works

Existing research has explored both external and internal factors influencing careless responses in online surveys. External factors include extrinsic motivation, social contact, and environmental distractions. For instance, Gadiraju et al [16] found that participants recruited through crowdsourcing platforms frequently use strategies to minimize time and effort in exchange for compensation. As survey duration increases, participants’ motivation and attention tend to decline, significantly elevating the risk of CR. In addition, Johnson [17] argued that the lack of direct interaction between respondents and administrators, as well as physical distance in online surveys, may reduce participants’ sense of responsibility, leading to more careless responses. Environmental distractions can further exacerbate CR [18].

Internal factors, such as participants’ intrinsic motivation, interest, and personality traits, also play a significant role in CR. For example, participants with low survey interest are more likely to exhibit careless responses in lengthy surveys compared to shorter ones [19]. As survey duration increases, participants’ motivation diminishes, further elevating the probability of careless responses [15]. In addition, Maniaci and Rogge [20] found that self-reported agreeableness, conscientiousness, and openness to experience were negatively correlated with CR. Bowling et al [21] reported that acquaintance-reported agreeableness, conscientiousness, emotional stability, and extraversion were negatively correlated with CR. Similarly, Meade et al [13] found that self-reported agreeableness, friendliness, and emotional stability were negatively correlated with CR in a sample of undergraduate students. McKay [22] further revealed that benevolent personality traits (eg, self-esteem) were negatively correlated with CR, while malevolent traits (eg, interpersonal coldness and sensation-seeking) were positively correlated.

### Research Gaps

While existing research has extensively explored external and internal factors influencing CR in online surveys, these studies have primarily focused on nonpsychological screening contexts. Unlike general questionnaires, psychological screening instruments often include intrusive or sensitive items (eg, “Have you had suicidal thoughts in the past two weeks?”), which may trigger defensive avoidance or emotional arousal in respondents. As a result, the factors contributing to CR in psychological screening questionnaires may differ significantly from those in general online surveys, highlighting the need for further investigation into this unique context.

Moreover, existing research has produced inconsistent findings regarding the same factors influencing CR, potentially due to the heterogeneous effect of questionnaire type. Most studies analyze data from a single questionnaire type, whereas mental health screening questionnaires encompass diverse forms, such as the Patient Health Questionnaire-9 (PHQ-9) [23], Generalized Anxiety Disorder-7 Scale (GAD-7) [24], Insomnia Severity Index (ISI) [25], and Perceived Stress Scale (PSS) [26]. Thus, the same factors may yield different conclusions across different psychological screening instruments. However, few studies have examined response behaviors across multiple psychological screening questionnaires within the same participant group.

### Objective

This study aims to explore the factors influencing CR in online mental health screening questionnaires and assess how the effect of these factors varies across different types of psychological screening instruments. The findings can improve the validity of psychological screening outcomes, ultimately advancing mental health services.

## Methods

### Study Design and Participants

This cross-sectional study is based on observational data collected through an online mental health screening initiative conducted by Xinxiang Medical University during the COVID-19 outbreak (February 27-March 17, 2021) [27], which aimed to assess psychological distress among students to inform campus mental health support services. Eligible participants were currently enrolled undergraduate or graduate students at Xinxiang Medical University who voluntarily participated in the screening and provided informed consent for future research purposes.

### Data Collection

The data were collected through a large-scale online psychological screening project conducted at Xinxiang Medical University during the COVID-19 pandemic [27]. The project aimed to assess the mental health status of college students. The study used a web-based application accessible via QR codes or URLs, allowing participants to complete the survey voluntarily on their personal devices without restrictions on time or location, although all data collection was confined to the campus due to pandemic restrictions. Participants included 24,367 undergraduate and graduate students, representing a convenience sample recruited through class-wide notifications distributed by counselors. The questionnaire comprised standard psychological screening instruments administered in a fixed sequence: the PHQ-9, GAD-7, PSS, and ISI, along with demographic items. These instruments maintained their original scoring systems. Specifically, the PHQ-9 consists of 9 items scored from 0 to 3 (total score range 0‐27), the GAD-7 includes 7 items scored from 0 to 3 (range 0‐21), the PSS comprises 14 items scored from 0 to 4 (range 0‐56), and the ISI contains 7 items scored from 0 to 4 (range 0‐28), with each question’s response time recorded to the nearest 0.01 seconds precision using timestamps. Prior to completing the psychological screening instruments, all participants were required to report demographic information, including age, sex, education, smoking, and drinking. Each variable was collected through a structured response format with predefined options. The anonymized dataset was published on Zenodo in comma-separated values format. To ensure data quality, the study implemented multiple controls: participants were required to complete all items (75 participants were excluded for missing responses), and response times were later screened for outliers using the median absolute deviation.

### Variables and Measurements

CR was measured using the Page Time Index (PTI), which captures the proportion of items answered in less than 2 seconds per item—a widely accepted threshold for detecting CR [28-30]. While PTI is typically computed using page-level timestamps (eg, average time per item on a multi-item page), our dataset provided item-level response time, enabling more precise measurement. For each clinical item, we recorded the response duration from item presentation to answer submission. Items completed in less than 2 seconds were coded as “1” (careless), and those completed in 2 seconds or more were coded as “0” (careful). The CR was then calculated as the average of these binary indicators across all items, resulting in a continuous score ranging from 0 to 1 that reflects the proportion of careless responses.

The independent variables included sex, age, education, smoking, and drinking. All variables were directly reported by participants through the demographic questions, with sex as a binary variable and age as a continuous variable. Education, smoking, and drinking were categorical variables. All variables were based on prior empirical evidence. Specifically, sex was included because previous studies have shown differences in response patterns between males and females [15], while age was incorporated due to its established relationship with attention span and survey engagement [31]. Education level was selected, as higher educational attainment is linked to more conscientious responding [32]. Behavioral factors such as smoking [17] and drinking [31] were included based on research suggesting these behaviors may influence response effort and impulsiveness.

### Statistical Analysis

This study used secondary data, involving 24,367 college students. As the data were collected for screening rather than research purposes, the sample size was determined by the organizers of the original screening initiative rather than by formal power calculations. To evaluate whether this sample size was sufficient for our analytical objectives, we conducted post hoc power analyses using G*Power 3.1, developed by Faul et al [33,34]. With a sample size of 24,367 and 5 predictors, the achieved power was 100% to detect small effects (f²=0.02, *α*=.05). Sensitivity analyses confirmed 100% power for even smaller effects (f²=0.005). Therefore, the current sample size meets the statistical power requirements. To examine the factors associated with CR in online psychological assessments, we used a multiple linear regression model using SPSS Statistics (version 27.0; IBM). The multiple linear regression model is specified as follows:



(1)
$$\begin{array}{rl} CR=\; & \beta_{0}+\beta_{1}\cdot \text{Sex}+\beta_{2}\cdot \text{Age}+\beta_{3}\cdot {\text{Education}}_{\text{bachelor}}\\ +\beta_{4}\cdot {\text{Education}}_{\text{master}}+\beta_{5}\cdot {\text{Education}}_{\text{doctor}}\\ +\beta_{6}\cdot {\text{Smoking}}_{\text{former}}+\beta_{7}\cdot {\text{Smoking}}_{\text{occasional}}+\beta_{8}\cdot {\text{Smoking}}_{\text{current}}\\ +\beta_{9}\cdot {\text{Drinking}}_{\text{former}}+\beta_{10}\cdot {\text{Drinking}}_{\text{occasional}}+\beta_{11}\cdot {\text{Drinking}}_{\text{current}}\\ +\varepsilon \end{array}$$

The dependent variable was CR, operationalized as a continuous score ranging from 0 to 1. Independent variables included sex (binary: male or female), age (continuous), education (categorical: bachelor’s, master’s, or doctoral degree, with associate degree as the reference group), smoking (categorical: former, occasional, or current smoker, with never smoker as the reference group), and drinking (categorical: former, occasional, or current drinker, with never drinker as the reference group). The selection of these variables was based on theoretical considerations and prior empirical findings [15,17,31,32], which suggest their potential associations with attention, cognitive effort, and behavioral compliance in survey settings.

To facilitate statistical analysis, we converted all categorical variables into dummy variables and included them in the multiple linear regression model. These dummy variables allow us to examine the unique effect of each category while controlling for other covariates. Given the relatively small number of predictors (5 variables) and their strong theoretical justification, we opted not to use stepwise selection methods, instead retaining all specified variables to avoid potential overfitting and maintain theoretical coherence [35]. Multicollinearity diagnostics using variance inflation factors (all <2.0) confirmed the absence of problematic correlations among predictors.

### Ethical Considerations

The original data collection received ethical approval from the Ethics Committee of Xinxiang Medical College of Henan Province (XYLL-2020235) [27]. The dataset used in this study is publicly available, fully anonymized, and contains no personally identifiable information. Therefore, in accordance with prevailing guidelines for exempt research involving secondary anonymized data, no additional ethical review was required. Informed consent was obtained online from all participants prior to survey administration, including agreement for data sharing and dissemination for scientific research. This study ensured adherence to all necessary ethical considerations and informed consent procedures. To protect participants’ personal information, all data collected in the original screening were fully anonymized before analysis [27]. No explicit incentives or compensation were provided to participants, as the screening was a voluntary public health service intended to benefit students’ mental health.

## Results

### Participants

The study initially collected 24,367 responses, from which 131 participants were excluded: 75 for missing values and 56 for implausible age entries. This yielded a final analytical sample of 24,236 participants, as detailed in Table 1. Female students predominated (64.0%, n=15,518), and most participants (84.7%, n=20,516) fell within the age range of 16‐22 years. Educational attainment showed a clear hierarchical distribution, with bachelor’s degree holders constituting 91.8% (n=22,259) of the sample, while graduate students were comparatively underrepresented (master’s: 2.8%, n=666 and doctoral: 0.1%, n=33). Most participants reported never smoking (92.7%, n=22,468) or consuming alcohol (67.5%, n=16,372), consistent with general population trends among Chinese university students.

**Table 1.** Demographic and descriptive characteristics of survey participants.

Number of respondents	Value
All, N (%)	24,236 (100)
Sex, n (%)	
Male	8718 (36)
Female	15,518 (64)
Age (years), n (%)	
16‐22	20,516 (84.7)
23‐29	3643 (15)
30‐35	77 (0.3)
Education, n (%)	
Associate degree	1278 (5.3)
Bachelor’s degree	22,259 (91.8)
Master’s degree	666 (2.8)
Doctoral degree	33 (0.1)
Smoking, n (%)	
Never smokes	22,468 (92.7)
Former smoker (cumulative smoking >10 packs), but not in the past year	189 (0.8)
Occasional smoker (cumulative smoking <10 packs)	1039 (4.3)
Current smoker (cumulative smoking >10 packs)	540 (2.2)
Drinking, n (%)	
Never drinks	16,372 (67.5)
Drank in the past (more than once a week), but not in the past year	282 (1.2)
Drinks occasionally (less than once a week)	7344 (30.3)
Current regular drinker (more than once a week)	238 (1)

### Variable Definitions

This study examines the following independent variables: sex, age, education, smoking, and drinking. Detailed variable coding is provided in Textbox 1.

Textbox 1. **Variable definitions.**

**Variable and assignment instructions**

- Sex: 0=male; 1=female.
- Age: age in years.
- Education: 1=associate degree; 2=bachelor’s degree; 3=master’s degree; and 4=doctoral degree.
- Smoking: 1=never smokes; 2=former smoker (cumulative smoking >10 packs), but not in the past year; 3=occasional smoker (cumulative smoking <10 packs); and 4=current smoker (cumulative smoking >10 packs).
- Drinking: 1=never drinks; 2=drank in the past (more than once a week), but not in the past year; 3=drinks occasionally (less than once a week); and 4=current regular drinker (more than once a week).

### Regression Results of Careless Response

To ensure data quality, we implemented a rigorous outlier removal protocol, excluding responses with completion times exceeding 3 SDs from the mean. This procedure resulted in slightly varied final sample sizes across instruments: PHQ-9 (n=23,984), PSS (n=24,133), ISI (n=24,152), and GAD-7 (n=24,165). The multiple linear regression results for the PHQ-9, PSS, ISI, and GAD-7 scales are presented in Tables 2-5.

**Table 2.** Regression results of careless reactions on the Patient Health Questionnaire-9 scale.

Variable	Standardized coefficients *β* (95% CI)	*P* value
Sex (reference=male)		
Female	−0.172 (−0.104 to −0.089)	<.001
Age (years)	−0.036 (−0.007 to −0.003)	<.001
Education (reference=associate degree)		
Bachelor’s degree	0.057 (0.041 to 0.071)	<.001
Master’s degree	0.098 (0.136 to 0.188)	<.001
Doctoral degree	0.028 (0.109 to 0.291)	<.001
Smoking (reference=never smokes)		
Former smoker, but not in the past year	0.001 (−0.035 to 0.043)	.84
Occasional smoker	0.002 (−0.015 to 0.02)	.75
Current smoker	−0.022 (−0.064 to −0.016)	.001
Drinking (reference=never drinks)		
Drank in the past, but not in the past year	−0.046 (−0.147 to −0.084)	<.001
Drinks occasionally	−0.163 (−0.103 to −0.087)	<.001
Current regular drinker	−0.035 (−0.132 to −0.060)	<.001

**Table 3.** Regression results of careless reactions on the Perceived Stress Scale.

Variable	Standardized coefficient *β* (95% CI)	*P* value
Sex (reference=male)		
Female	−0.234 (−0.162 to −0.144)	<.001
Age (years)	−0.01 (−0.004 to 0.001)	.15
Education (reference=associate degree)		
Bachelor’s degree	0.028 (0.014 to 0.049)	<.001
Master’s degree	0.061 (0.087 to 0.147)	<.001
Doctoral degree	0.028 (0.128 to 0.339)	<.001
Smoking (reference=never smokes)		
Former smoker, but not in the past year	0.018 (0.019 to 0.109)	.005
Occasional smoker	0.019 (0.01 to 0.050)	.003
Current smoker	−0.003 (−0.034 to 0.022)	.68
Drinking (reference=never drinks)		
Drank in the past, but not in the past year	−0.018 (−0.088 to −0.015)	.006
Drinks occasionally	−0.112 (−0.086 to −0.067)	<.001
Current regular drinker	−0.015 (−0.088 to −0.005)	.03

**Table 4.** Regression results of careless reactions on the Insomnia Severity Index scale.

Variable	Standardized coefficients *β* (95% CI)	*P* value
Sex (reference=male)		
Female	−0.207 (−0.13 to −0.114)	<.001
Age (years)	−0.036 (−0.007 to −0.003)	<.001
Education (reference=associate degree)		
Bachelor’s degree	0.065 (0.051 to 0.083)	<.001
Master’s degree	0.089 (0.127 to 0.182)	<.001
Doctoral degree	0.031 (0.144 to 0.335)	<.001
Smoking (reference=never smokes)		
Former smoker, but not in the past year	0.008 (−0.015 to 0.065)	.23
Occasional smoker	0.007 (−0.009 to 0.027)	.32
Current smoker	−0.003 (−0.031 to 0.019)	.64
Drinking (reference=never drinks)		
Drank in the past, but not in the past year	−0.038 (−0.134 to −0.068)	<.001
Drinks occasionally	−0.135 (−0.091 to −0.074)	<.001
Current regular drinker	−0.033 (−0.131 to −0.057)	<.001

**Table 5.** Regression results of careless reactions on the Generalized Anxiety Disorder-7 Scale.

Variable	Standardized coefficient *β* (95% CI)	*P* value
Sex (reference=male)		
Female	−0.177 (−0.108 to −0.093)	<.001
Age (years)	−0.053 (−0.009 to −0.005)	<.001
Education (reference=associate degree)		
Bachelor’s degree	0.056 (0.04 to 0.07)	<.001
Master’s degree	0.091 (0.125 to 0.178)	<.001
Doctoral degree	0.021 (0.064 to 0.249)	.001
Smoking (reference=never smokes)		
Former smoker, but not in the past year	0.014 (0.005 to 0.083)	.03
Occasional smoker	0.002 (−0.015 to 0.020)	.80
Current smoker	−0.014 (−0.051 to −0.002)	.03
Drinking (reference=never drinks)		
Drank in the past, but not in the past year	−0.040 (−0.133 to −0.069)	<.001
Drinks occasionally	−0.144 (−0.094 to −0.077)	<.001
Current regular drinker	−0.030 (−0.12 to −0.048)	<.001

Table 2 shows that female students demonstrated lower levels of CR than male students when completing the PHQ-9 (*P*<.001). Older participants showed significantly lower levels of CR (*P*<.001). Participants with higher education levels had significantly higher levels of CR compared to the associate degree group (*P*<.001). Compared to the nonsmokers, current smokers demonstrated lower levels of CR (*P*=.001). Participants with a history of alcohol consumption also demonstrated lower levels of CR than those who never drank (*P*<.001).

Table 3 reveals that when completing the PSS questionnaire, female students again demonstrated lower levels of CR than male students (*P*<.001). Participants with bachelor’s (*P*<.001), master’s (*P*<.001), or doctoral degrees (*P*<.001) showed higher levels of CR compared to those with associate degrees. Both former smokers (*P*=.005) and occasional smokers (*P*=.003) were more prone to CR than nonsmokers. Participants with a history of alcohol consumption—whether past drinkers (*P*=.006), occasional drinkers (*P*<.001), or frequent drinkers (*P*=.03)—demonstrated lower levels of CR than those who never drank.

Table 4 reveals that female undergraduates demonstrated lower levels of CR than their male counterparts when completing the ISI (*P*<.001). For each unit increase in age, the level of CR on the ISI scale significantly decreased (*P*<.001). That is, older individuals were less prone to CR. Relative to associate degree holders, progressively higher education levels—bachelor’s (*P*<.001), master’s (*P*<.001), and doctoral (*P*<.001)—showed higher levels of CR. All drinking categories—former drinkers (*P*<.001), occasional drinkers (*P*<.001), and frequent drinkers (*P*<.001)—demonstrated lower levels of CR relative to nondrinkers.

Table 5 indicates a pronounced sex difference emerged, with female students demonstrating lower levels of CR than males when completing the GAD-7 (*P*<.001). Older participants showed significantly lower levels of CR (*P*<.001). Relative to associate degree holders, participants with bachelor’s (*P*<.001), master’s (*P*<.001), and doctoral degrees (*P*=.001) consistently exhibited higher levels of CR. Former smokers showed significantly higher levels of CR compared to their nonsmoking peers (*P*=.03), and current smokers demonstrated lower levels of CR (*P*=.03). All categories of drinkers—including former drinkers (*P*<.001), occasional drinkers (*P*<.001), and regular drinkers (*P*<.001)—demonstrated lower levels of CR than nondrinkers.

Multimedia Appendix 1 presents several consistent and divergent patterns in CR across the 4 psychological scales. Most notably, female participants demonstrated lower levels of CR than males in all questionnaires (*P*<.001 for all scales). Age was associated with lower levels of CR on the PHQ-9 (*P*<.001), ISI (*P*<.001), and GAD-7 (*P*<.001), but not on the PSS (*P*=.15). Educational attainment consistently demonstrated higher levels of CR, with advanced degree holders showing higher levels than associate degree holders across all measures. Smokers exhibited varied patterns, with current smokers demonstrating lower levels of CR on the PHQ-9 and GAD-7, but occasional smokers demonstrating higher levels of CR on the PSS than nonsmokers. Interestingly, college students with a history of drinking demonstrated lower levels of CR across all scales compared to those who had never consumed alcohol.

### Pattern Identification

**Figure 1.**
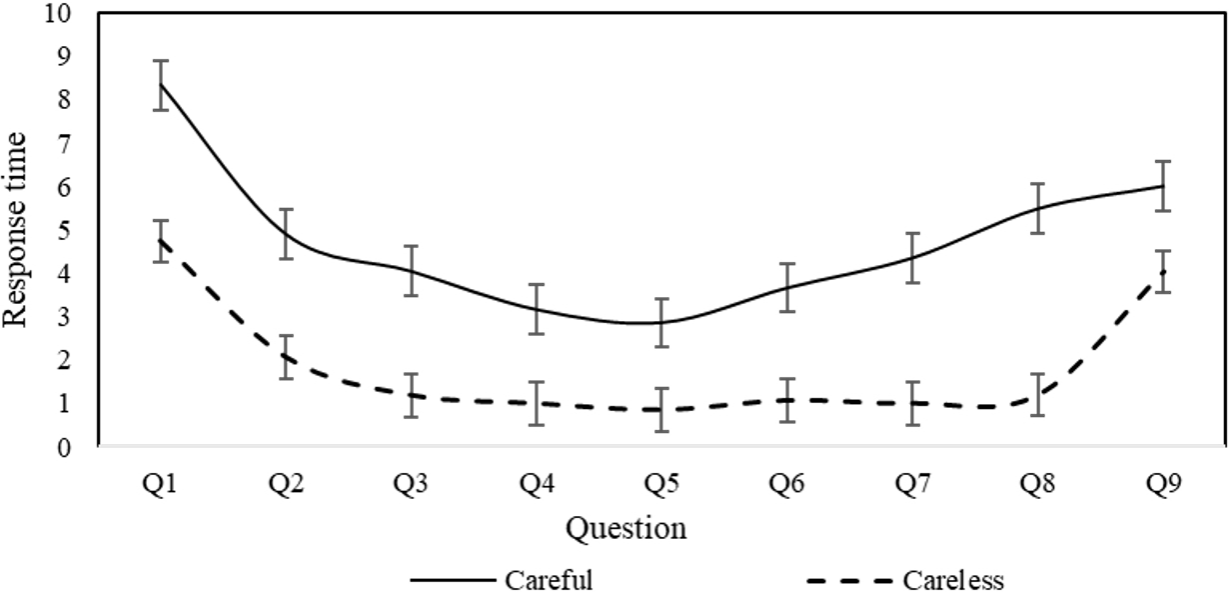
Response time for individual questions of the Patient Health Questionnaire-9.

**Figure 2.**
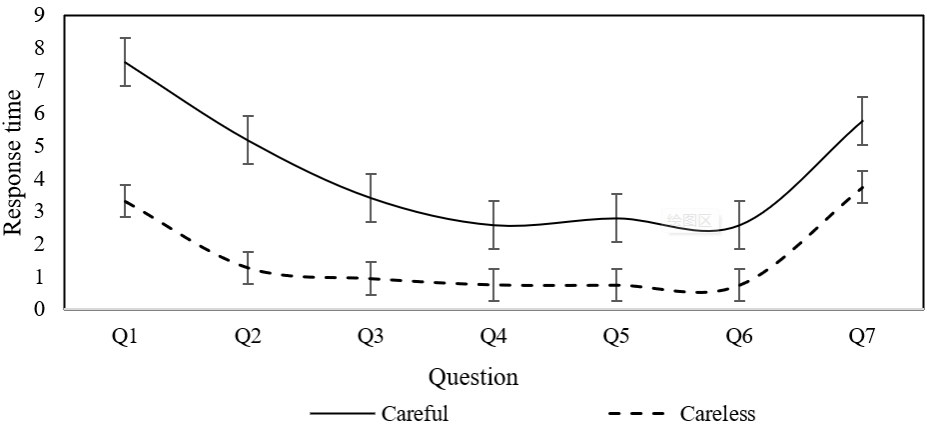
Response time for individual questions of the Generalized Anxiety Disorder-7 Scale.

**Figure 3.**
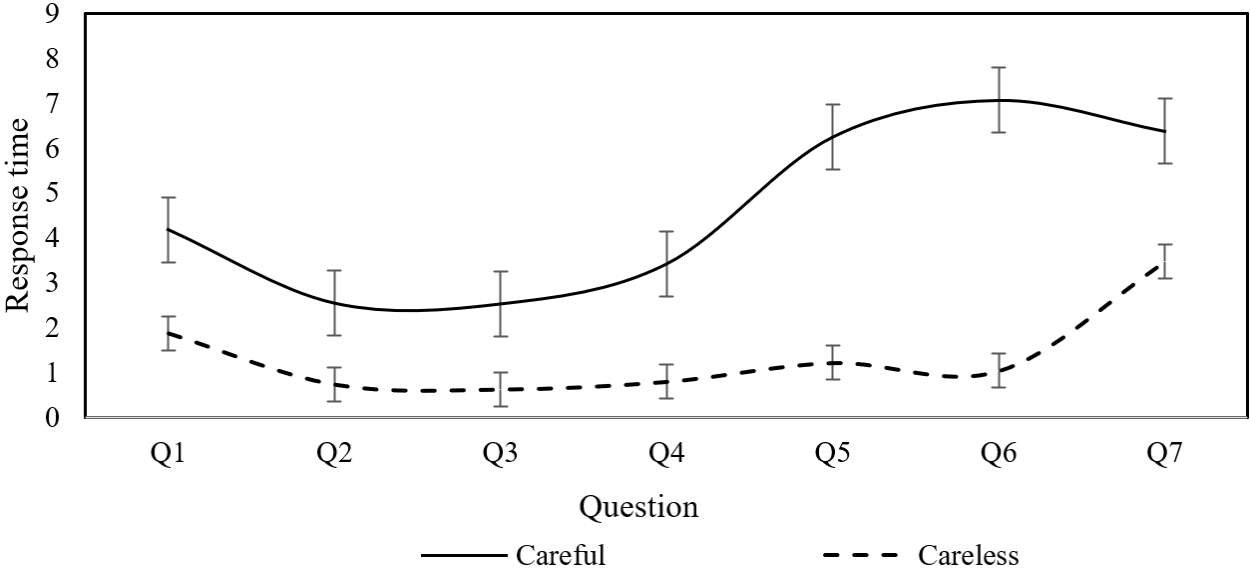
Response time for individual questions of the Insomnia Severity Index.

**Figure 4.**
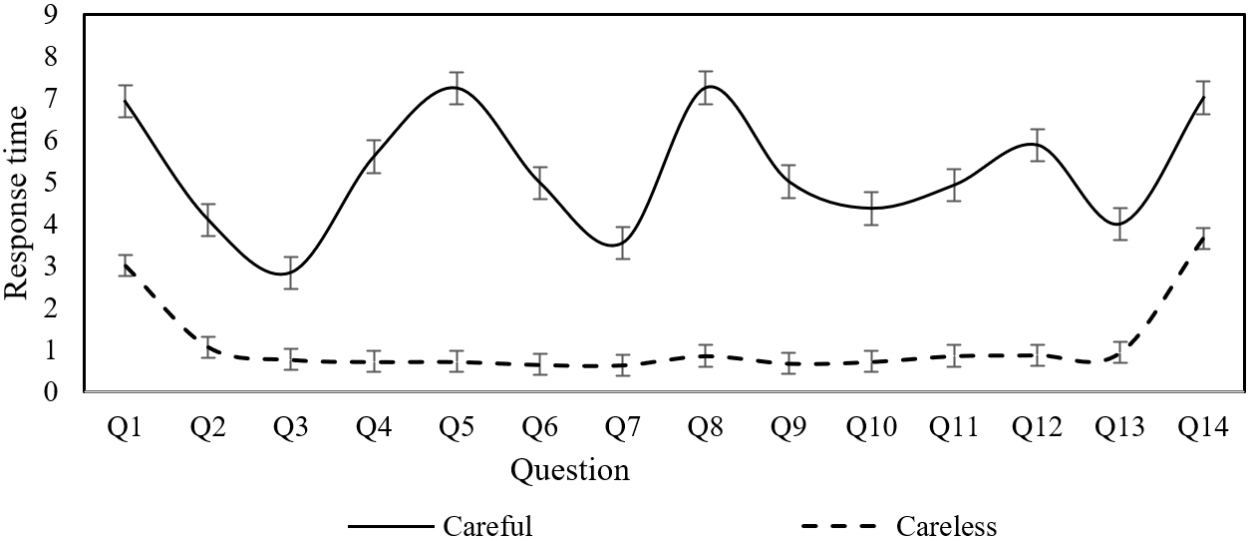
Response time for individual questions of the Perceived Stress Scale.

While the study has identified factors influencing CR in mental health screening questionnaires, the specific behavioral patterns underlying careless responses remain unclear. Understanding these patterns is crucial for improving questionnaire design and ultimately enhancing the validity and reliability of psychological assessments. To explore this, based on Erdem Kara’s study [30], we classified users whose PTI exceeded 0.67 seconds as careless respondents. The average response times with error bars for each question were calculated separately for careless and careful responders across the PHQ-9, PSS, ISI, and GAD-7 scales. The findings are presented in Figures 1-4.

As illustrated in Figures1  2, both careless and careful responders exhibit similar trends in response time per question across the PHQ-9 and GAD-7 scales. However, careless responders demonstrate significantly shorter response times compared to careful responders. This pattern suggests that careless responders tend to skim through questions rapidly, without engaging in thorough reading or thoughtful consideration. In contrast, Figures3  4 reveal distinct behaviors for the ISI and PSS scales: careful responders display variability in response times per question, while careless responders maintain nearly constant response times. This finding suggests that careless responders may skip reading the questions altogether, opting instead to select answers automatically.

This behavior can be attributed to the varying levels of importance participants place on different psychological conditions. For the PHQ-9 and GAD-7 scales, which assess depression and anxiety symptoms, even careless responders may still skim through the questions due to the perceived significance of these conditions. In contrast, for the ISI and PSS scales, which measure insomnia severity and perceived stress, participants may perceive these conditions as less critical. As a result, they may disregard the questions entirely, selecting answers to complete the task with minimal effort. This suggests that perceived importance influences the depth of engagement, with less salient conditions such as stress and insomnia receiving significantly less attention.

## Discussion

### Principal Findings

The study found that female participants demonstrated lower levels of careless responding than male participants when filling out psychological screening questionnaires. This is consistent with previous research findings [31]. This phenomenon can be explained from the perspectives of emotional expression differences and social expectations. First, women demonstrated stronger emotional expression and empathy [36], enabling them to better understand the emotional nuances of the questionnaire and take mental health–related tasks more seriously. Second, social expectation theory suggests that women are more inclined to conform to social norms [37], particularly in tasks involving emotions and mental health.

Older participants demonstrated significantly lower levels of CR on the PHQ-9, ISI, and GAD-7, whereas no significant age-related difference emerged for the PSS. This pattern aligns with previous findings, such as those of Carstensen and DeLiema [38], who suggest that older adults exhibit enhanced emotional regulation and task focus—traits that may reduce CR on symptom-focused questionnaires. The absence of age effects for the PSS could stem from its unique assessment of perceived stress, as the PSS measures acute stress responses to daily challenges rather than chronic emotional states, potentially eliciting similar levels of attention across age groups.

Individuals with higher education levels showed more careless response behaviors when filling out psychological screening questionnaires. This finding contradicts conventional wisdom, as higher education is typically associated with greater conscientiousness in task completion. The tendency may stem from several factors. First, their deeper awareness of the stigma surrounding mental illness [39,40] could lead them to avoid negative labels by responding carelessly. Second, perfectionistic tendencies among this group [41,42] may make them overly sensitive to questionnaire items, potentially prompting careless responses.

Smokers exhibited varied patterns, with current smokers demonstrating lower levels of CR to the PHQ-9 and GAD-7, but occasional smokers showing higher levels of CR to the PSS than nonsmokers.

Drinkers consistently demonstrated lower levels of CR than nondrinkers. A potential explanation is that individuals with a history of drinking may be more attuned to their mental state, leading them to complete the questionnaires more carefully.

When filling out the PHQ-9 and GAD-7 questionnaires, careless responders were characterized by quickly skimming through the questions, whereas when filling out the PSS and ISI questionnaires, careless responders appeared to skip some questions and directly select answers. These findings provide important references for questionnaire designers and distributors, suggesting that targeted intervention measures should be taken.

### Comparison With Previous Work

Previous studies primarily examined the influence of external factors (eg, external motivation [16], social contact [17], and environmental distractions [18]) and internal factors (eg, intrinsic motivation [19], interest [15], and personality traits [20,22,32,43]) on careless responses. In contrast, this study delves deeper into how individual factors manifest differently across various types of psychological screening questionnaires. For instance, while prior research generally suggested that individuals with higher education levels are more likely to complete tasks conscientiously [44,45], this study found that highly educated individuals exhibited greater careless response behaviors when filling out psychological screening questionnaires. This discrepancy may be attributed to the intrusive nature of certain questionnaire items—highly educated individuals, driven by strong self-esteem, may be reluctant to disclose mental health concerns. In addition, this study identified variations in how age, smoking, and drinking behaviors influence careless responses across different questionnaire types. These findings diverge from previous conclusions, highlighting the need to account for the interaction between questionnaire type and individual characteristics when analyzing careless response behaviors.

### Clinical and Practical Implications

Our findings underscore critical implications for improving mental health screening practices, particularly regarding respondents’ differential engagement across questionnaire types. First, the lower conscientiousness observed for PSS (stress) and ISI (insomnia) measures compared to depression and anxiety scales suggests that many participants may underestimate the clinical significance of stress and sleep disturbances. To address this, we strongly recommend incorporating clear educational messaging during questionnaire administration that emphasizes the serious health consequences of unmanaged stress and insomnia (eg, increased risks for cardiovascular disease, diabetes, and cognitive decline). Second, for mental disorder screening, our results indicate the need for additional validity checks when assessing highly educated populations, who may demonstrate greater careless response potentially due to stigma concerns. Third, older adults showed lower levels of CR with depression, sleep, and anxiety items, highlighting a critical need for early mental health education. Proactive interventions, such as psychoeducation campaigns targeting younger demographics, could help mitigate the long-term consequences of untreated symptoms, which often compound with age.

### Limitations and Future Directions

This study has several limitations that could be studied in future work. First, our operationalization of CR relied on response time, which may not capture all forms of inattentive responding. Future work should combine response time metrics with other validated approaches such as eye-tracking methodologies and postsurvey self-reports of engagement. Second, the homogeneous sample—college students from a single Chinese medical university—raises concerns about generalizability. Future work could examine whether these conclusions generalize to diverse domains, such as international contexts with varying cultural norms. Third, while we controlled for major demographic and behavioral factors, unmeasured variables (eg, personality traits) may influence CR.

### Conclusion

This study analyzed data from college students who completed 4 common psychological screening questionnaires online, identifying the individual factors that influence careless response behaviors and their heterogeneous effects across different questionnaire types. The findings indicate that sex, age, education level, smoking, and drinking habits significantly impact CR, with these effects differing across screening tools. Moreover, the study revealed distinct patterns of careless responses depending on the questionnaire type. When completing the PHQ-9 and GAD-7, careless responders tended to skim through questions quickly, whereas in the PSS and ISI, they were more likely to skip questions and select answers without careful consideration.

These findings offer important guidance for both clinical practice and future research. For clinicians administering online mental health screenings, the results suggest implementing questionnaire-specific quality control measures, such as response time monitoring for PHQ-9 and GAD-7 assessments, and mandatory question-answering features for PSS and ISI measures. The identified demographic risk factors (eg, male sex and higher education) can help target these interventions to high-risk groups. For researchers, the study highlights the need to (1) develop more sophisticated detection algorithms that account for these instrument-specific response patterns, (2) investigate the psychological mechanisms underlying the observed demographic differences, and (3) examine whether these findings generalize to clinical populations and other cultural contexts.

## Supplementary material

10.2196/70451 Multimedia Appendix 1 Regression results of careless responses across different questionnaires.

## Abbreviations

CR: careless responding
GAD-7: Generalized Anxiety Disorder-7 Scale
ISI: Insomnia Severity Index
PHQ-9: Patient Health Questionnaire-9
PSS: Perceived Stress Scale
PTI: Page Time Index
WHO: World Health Organization
