# Supplementary material for: The Impact of Individual Factors on Careless Responding Across Different Mental Disorder Screenings: Cross-Sectional Study
Source: J Med Internet Res. 2025 Jul 31;27:e70451. doi: 10.2196/70451 (PMC12323810; doi:10.2196/70451)
Supplement: Multimedia Appendix 1 [file jmir-v27-e70451-s001.docx]

**Table 7.** Regression results of careless responses across different questionnaires.

| Variable | PHQ-9:  coefficient  (P value) | PSS:  coefficient  (P value) | ISI:  coefficient  (P value) | GAD-7:  coefficient  (P value) |
| --- | --- | --- | --- | --- |
| Sex (reference=male) | | | | |
| Female | −0.172  (<.001) | −0.234  (<.001) | −0.207  (<.001) | −0.177  (<.001) |
| Age (years) | −0.036  (<.001） | −0.01  (.15) | −0.036  (<.001) | −0.053  (<.001) |
| Education (reference=associate degree) | | | | |
| Bachelor’s degree | 0.057  (<.001) | 0.028  (<.001) | 0.065  (<.001) | 0.056  (<.001) |
| Master’s degree | 0.098  (<.001) | 0.061  (<.001) | 0.089  (<.001) | 0.091  (<.001) |
| Doctoral degree | 0.028  (<.001) | 0.028  (<.001) | 0.031  (<.001) | 0.021  (.001) |
| Smoking (reference=never smokes) | | | | |
| Former smoker, but not in the past year | 0.001  (.84) | 0.018  (.005) | 0.008  (.23) | 0.014  (.03) |
| Occasional smoker | 0.002  (.75) | 0.019  (.003) | 0.007  (.32) | 0.002  (.80) |
| Current smoker | −0.022  (.001) | −0.003  (.68) | −0.003  (.64) | −0.014  (.03) |
| Drinking (reference=never drinks) | | | | |
| Drank in the past, but not in the past year | −0.046  (<.001) | −0.018  (.006) | −0.038  (<.001) | −0.040  (<.001) |
| Drinks occasionally | −0.163  (<.001) | −0.112  (<.001) | −0.135  (<.001) | −0.144  (<.001) |
| Current regular drinker | −0.035  (<.001) | −0.015  (.03) | −0.033  (<.001) | −0.030  (<.001) |
